# The Survival of Human Intervertebral Disc Nucleus Pulposus Cells under Oxidative Stress Relies on the Autophagy Triggered by Delphinidin

**DOI:** 10.3390/antiox13070759

**Published:** 2024-06-23

**Authors:** Md Entaz Bahar, Jin Seok Hwang, Trang Huyen Lai, June-Ho Byun, Dong-Hee Kim, Deok Ryong Kim

**Affiliations:** 1 Department of Biochemistry and Convergence Medical Sciences, Institute of Medical Science, College of Medicine, Gyeongsang National University, Jinju 52727, Republic of Korea; mdentaz87@gnu.ac.kr (M.E.B.); cloud8104@naver.com (J.S.H.); tranghuyen20493@gmail.com (T.H.L.); 2 Department of Oral and Maxillofacial Surgery, Institute of Medical Science, College of Medicine, Gyeongsang National University Hospital, Gyeongsang National University, Jinju 52727, Republic of Korea; surbyun@gnu.ac.kr; 3 Department of Orthopaedic Surgery, Institute of Medical Science, College of Medicine, Gyeongsang National University Hospital, Gyeongsang National University, Jinju 52727, Republic of Korea

**Keywords:** delphinidin, IVDD, oxidative stress, senescence, apoptosis, ECM degradation, autophagy

## Abstract

Delphinidin (Delp), a natural antioxidant, has shown promise in treating age-related ailments such as osteoarthritis (OA). This study investigates the impact of delphinidin on intervertebral disc degeneration (IVDD) using human nucleus pulposus cells (hNPCs) subjected to hydrogen peroxide. Various molecular and cellular assays were employed to assess senescence, extracellular matrix (ECM) degradation markers, and the activation of AMPK and autophagy pathways. Initially, oxidative stress (OS)-induced hNPCs exhibited notably elevated levels of senescence markers like p53 and p21, which were mitigated by Delp treatment. Additionally, Delp attenuated IVDD characteristics including apoptosis and ECM degradation markers in OS-induced senescence (OSIS) hNPCs by downregulating MMP-13 and ADAMTS-5 while upregulating COL2A1 and aggrecans. Furthermore, Delp reversed the increased ROS production and reduced autophagy activation observed in OSIS hNPCs. Interestingly, the ability of Delp to regulate cellular senescence and ECM balance in OSIS hNPCs was hindered by autophagy inhibition using CQ. Remarkably, Delp upregulated SIRT1 and phosphorylated AMPK expression while downregulating mTOR phosphorylation in the presence of AICAR (AMPK activator), and this effect was reversed by Compound C, AMPK inhibitor. In summary, our findings suggest that Delp can safeguard hNPCs from oxidative stress by promoting autophagy through the SIRT1/AMPK/mTOR pathway.

## 1. Introduction

Age-related conditions such as osteoarthritis (OA) and intervertebral disc degeneration (IVDD) are closely associated with oxidative stress (OS). In particular, OS exacerbates during IVDD, significantly contributing to its progression [1]. Various pathophysiological pathways, including matrix metabolism, inflammation, apoptosis, autophagy, and disc cell senescence, play crucial roles in this progression [2]. The involvement of hydrogen peroxide (H_2_O_2_)-induced OS in the intervertebral disc (IVD) has been extensively studied due to its association with reactive oxygen species (ROSs). Hydrogen peroxide accelerates ROS generation and DNA damage in senescent nucleus pulposus (NP) cells within the IVD [3]. Senescent NP cells experience impaired proliferation due to accumulated cellular damage and permanent cell cycle arrest [4]. Stimulation of the ATM-Chk2-p53-p21-Rb and p16-Rb signaling pathways in NP cells induces premature cellular senescence [5,6]. Hydrogen peroxide can also induce a catabolic phenotype in senescent cells, characterized by increased expression of extracellular matrix-degrading enzymes (MMP-1, MMP-2, MMP-9, and ADAMTS-5), and by the decreased levels of their suppressors (TIMPs) and proteoglycans like aggrecan [5]. Moreover, H_2_O_2_ activates various signaling pathways, including p38 MAPKs, ERKs, and JNKs, and leads to the translocation of NF-κB and Nrf2 to the nucleus [5]. Additionally, hydrogen peroxide accelerates premature aging of cartilage endplate cells via the p53-p21-Rb pathway [7]. The marker p53 is associated with cellular aging [8]. Although various stressors contribute to cellular senescence in IVDD, significant knowledge gaps remain in this area, presenting opportunities for the development of therapeutic interventions for age-related IVDD.

OA and IVDD are also characterized by heightened inflammation, which is pivotal in the onset and progression of various diseases, influencing their pathophysiology [9]. Understanding the role of inflammation in OA and IVDD highlights the importance of focusing on inflammatory pathways for therapeutic intervention. Researchers are exploring anti-inflammatory drugs, including cytokine inhibitors and antioxidants, to slow the progression of these degenerative conditions and alleviate symptoms [10,11].

Autophagy, a cellular catabolic process, serves as the cellular quality control system by engulfing and breaking down damaged or dysfunctional cellular components. It acts as a fundamental process in maintaining cellular homeostasis and adapting to various environmental stresses. When cells encounter stressors like nutritional deficiency, viral infection, hypoxia, or oxidative and genotoxic stress, autophagy is upregulated to help mitigate the damage and restore cellular balance. Through this process, cells can recycle cytoplasmic components, including proteins and organelles, to generate energy and essential building blocks for survival [12]. Recent studies have shed light on the potential therapeutic implications of autophagy modulation in age-related degenerative diseases such as intervertebral disc degeneration and osteoarthritis [13,14]. While excessive autophagy may contribute to cellular dysfunction and disease progression, maintaining an optimal level of autophagic activity could potentially alleviate the pathological processes associated with these conditions. Thus, understanding the intricate regulation of autophagy and its role in disease pathogenesis holds promise for the development of novel therapeutic strategies targeting age-related degenerative disorders.

Anthocyanins exhibit heightened sensitivity to H_2_O_2_-induced oxidative stress and demonstrate superior capability in scavenging H_2_O_2_ compared to other phenolics, as reported in various studies [15,16]. Notably, the remarkable antioxidative activity of anthocyanins has led to their utilization as a distinct dietary supplement for free radical scavenging, thereby gaining prominence in addressing age-related ailments, including intervertebral disc degeneration [17]. Among these anthocyanins, delphinidin, renowned for its potent antioxidant properties, stands out as one of the most beneficial polyphenols. Previous research, including our own, has demonstrated the efficacy of delphinidin in protecting chondrocytes against oxidative stress associated with age-related conditions such as osteoarthritis (OA) [18,19]. Our previous findings indicated that delphinidin shields C28/I2 chondrocyte cells from ROS-induced apoptosis through the activation of Nrf2 and NF-κB pathways, while promoting protective autophagy mechanisms [18]. Despite delphinidin being a prominent bioactive compound within anthocyanins, the precise mechanisms underlying its protective effects against oxidative stress-induced senescence in age-related diseases remain inadequately characterized. We postulate that senescent cells may accumulate within the intervertebral disc with age and progressive disc degeneration. To elucidate the mechanisms underlying NP senescence, we employed H_2_O_2_ as an inducer of oxidative stress in human NP cells.

## 2. Materials and Methods

### 2.1. Human Nucleus Pulposus Cells Culture

Human nucleus pulposus cells (hNPCs) were purchased from ScienCell Research Laboratories (4800, ScienCell, Carlsbad, TX, USA) and cultured in Nucleus Pulposus Cell Medium (NPCM, ScienCell, Carlsbad, TX, USA) supplemented with 2% fetal bovine serum (FBS; ScienCell, Carlsbad, CA, USA), 1% penicillin/streptomycin (P/S; ScienCell, Carlsbad, CA, USA), and nucleus pulposus cell growth supplement (NPCGS; ScienCell, Carlsbad, CA, USA).

### 2.2. Hydrogen Peroxide (H_2_O_2_) Treatment

The hNPCs were cultured in 96-well plates at 1 × 10^4^ cells/well with 150 µL of NPCM in the presence of various doses of H_2_O_2_ (0 to 1000 µM) for 24 h to determine the cytotoxicity and inhibitory concentration (IC_50_) of H_2_O_2_. Cell viability was measured via a Cell Counting Kit test (CCK-8) (CK04-13; Dojindo, Kumamoto, Japan). The hNPCs (700 cells) were cultured in 6-well plates with 1.5 mL of NPCM in the presence of various doses of H_2_O_2_ (0, 110, 185, and 250 µM) for 24 h, followed by 7 days of recovery. Clonogenicity was measured by crystal violet staining assay.

### 2.3. Delphinidin Treatment

The hNPCs were cultured in 96-well plates at 1 × 10^4^ cells/well with 150 µL of NPCM in the presence of various doses of Delp (0 to 200 µM) at 24 h to determine the IC_50_ value of Delp. Cell viability was measured by the CCK-8 assay and crystal violet assay. The hNPCs were cultured in 96-well plates with 150 µL of NPCM in the presence of various doses of Delp (0, 2.5, 5, 10, 20, and 40 µM) or 5 mM of N-acetyl cysteine (NAC) at 12 and 24 h before 185 µM of H_2_O_2_ was added to determine the dose- and time-dependent effects of Delp. The hNPCs (700 cells) were cultured in 6-well plates with 1.5 mL of NPCM supplemented with 40 µM of Delp or 5 mM of NAC for 24 h before 185 µM of H_2_O_2_ was added for 24 h, followed by 7 days of recovery. Clonogenicity was determined by crystal violet staining assay.

### 2.4. Establishment of Oxidative Stress-Induced Senescence (OSIS)

H_2_O_2_ was employed to induce oxidative stress-induced senescence (OSIS), following previously established protocols with slight modifications (Figure S1) [20,21,22]. Primary human nucleus pulposus cells at a population doubling (PD) of 3.5 and a population doubling time (PDT) of 2.8 in passage 1 (P1) were utilized to establish the OSIS model. Treatment commenced 24 h post-seeding by incubating 3 × 10^5^ cells in a 6-well plate with 2 mL of culture medium. Cells were cultured under normal conditions (nOSIS-control) or treated with delphinidin (nOSIS-Delp) or subjected to H_2_O_2_ treatment (OSIS-control), and H_2_O_2_ treatment with delphinidin (OSIS-Delp). Briefly, cells were incubated for 24 h in 6-well plates with 2 mL of NPCM containing 2% FBS supplemented with or without 40 µM of delphinidin before exposure to 250 µM of H_2_O_2_ for 4 h in passage 2 (P2). In passage 3 (P3), cells were pretreated with or without 20 µM of delphinidin for 24 h after being split into a 1:3 ratio and exposed to 185 µM of H_2_O_2_ for 4 h, followed by 24 h of recovery. Similarly, in passage 4 (P4), cells pretreated with 10 µM of delphinidin for 24 h or not were exposed to 110 µM of H_2_O_2_ for 4 h, followed by 24 h of recovery. The senescence model was confirmed through SA-β-gal assay, Western blotting, and other assays. For mechanistic evaluation, OSIS cells were cultured with normal media, delphinidin (20 µM), chloroquine (10 µM CQ; autophagy inhibitor), 5-Aminoimidazole-4-carboxamide ribonucleotide (1 mM, AICAR; AMPK inducer), or compound C (10 µM, CC; AMPK inhibitor) for 72 h.

### 2.5. Cell Population Doubling Time (CPDT)

The hNPCs were seeded at a constant density (2500 cells/mL) on the surface of a 10 cm culture plate containing NPCM in a humidified incubator (37 °C, 5% CO_2_) for 10 days to assess the phenotypic and morphological alterations of the in vitro OSIS model. After seeding cells, the entire cultural medium was swapped out for a fresh one every three days. Trypan blue exclusion was used to assess the in vitro proliferative potential of hNPCs [23]. The numbers of cell population doubling (NCPD) [24] and cell population doubling time (CPDT) were then calculated based on the following equations (Equations (1) and (2)) [25]:

(1) $$\mathrm{NCPD}=3.322*\left(\mathrm{logNt}-\mathrm{logNi}\right)$$

(2) $$\mathrm{CPDT}=\left(t-\mathrm{ti}\right)/\mathrm{NCPD}$$

where Nt  and Ni are the cell numbers at a specific time point t (10 days) and at the initial time point ti (0 days), respectively.

### 2.6. Cell Viability Assay

Cell viability analysis was conducted using the CCK-8 assay. hNPCs were seeded in 96-well plates and incubated with varying concentrations of delphinidin, H_2_O_2_ alone, or a combination of both as indicated. Subsequently, 150 μL of media comprising 135 μL of NPCM and 15 μL of CCK-8 solution (CK04-13; Dojindo, Kumamoto, Japan) was added to each well and incubated for 120 min at 37 °C. Following incubation, absorbance was measured at 450 nm using a microplate reader.

### 2.7. Crystal Violet Staining Assay

The cells were allowed to proliferate for the crystal violet proliferation assay for the specified duration. Following this, the media were aspirated, and the cells were washed twice with 1X Phosphate Buffered Saline (PBS). Subsequently, a solution of 10% formalin in 1X PBS was added and incubated for 20 min at room temperature. After incubation, the formalin solution was removed, and 0.1% (*w*/*v*) crystal violet (# C0775-25G, Sigma, St. Louis, MO, USA) was added to each well and incubated for an additional 20 min at room temperature. The crystal violet solution was then discarded, and the plates were thoroughly washed with tap water, followed by air-drying at room temperature. For quantification purposes, 1% SDS was added to each well and incubated at room temperature for 30 min. The extracted solution was transferred to a 96-well plate and quantified by measuring the optical density (OD) at 590 nm.

### 2.8. Clonogenic Survival Assay

To evaluate the cells’ clonogenic survival capability, the exposure medium was aspirated, and cells were detached using trypsin and counted using a hemocytometer. Subsequently, cells were seeded at a density of approximately 700 cells in 2.0 mL of medium per well in 6-well plates. The plates were then incubated for 7 to 10 days at 37 °C with 5% CO_2_. Following the incubation period, clonogenic activity was assessed using the crystal violet proliferation assay described earlier.

### 2.9. Measurement of Reactive Oxygen Species (ROSs)

To measure reactive oxygen species levels, a dichlorodihydrofluorescein diacetate cellular ROS assay kit (ab113851) from Abcam was employed following the manufacturer’s instructions. hNPCs were cultured on Poly-l-lysine-coated 96-well plates, and dichlorodihydrofluorescein diacetate was added to the cultures 30 min before the end of the recovery period. Following washing steps, fluorescence intensity was measured at 485/535 nm using a microplate reader.

### 2.10. Senescence-Associated β-Galactosidase Staining (SA-β-Gal Staining)

To assess the aging process of hNPCs, a cellular senescence activity assay (catalog#: ENZ-KIT129, Enzo Life Sciences, Lausen, Switzerland) was conducted following the manufacturer’s protocol. Initially, cells were seeded at a density of 1 × 10^4^ cells per well in a 96-well plate and incubated for 72 h. Subsequently, the medium was aspirated, and after washing with PBS, hNPCs were lysed on ice using a lysis buffer and incubated at 4 °C for 5 min. The entire lysate was then transferred to a microcentrifuge tube and centrifuged at 14,000× *g* for 10 min at 4 °C to collect the supernatant as cell lysate. A quantity of 50 µL of the cell lysate was transferred to a 96-well plate, followed by the addition of 50 µL of freshly prepared assay buffer. The plate was then incubated at 37 °C, shielded from light, for 2 h. After incubation, 50 µL of the reaction mixture was transferred to a separate 96-well plate suitable for fluorescence measurement. The reaction was stopped by adding 200 µL of stop solution. Fluorescence intensity was measured using a fluorescence plate reader with excitation at 360 nm and emission at 465 nm.

### 2.11. Autophagy Flux Detection

The autophagic flux in response to Delp therapy was assessed using the CYTO-ID Autophagy detection kit (#ENZ-51031, Enzo Life Sciences, Lausen, Switzerland), according to the manufacturer’s protocol. Briefly, human nucleus pulposus cells (hNPCs) were cultured in a 37 °C incubator with 5% CO_2_ during the experimental period. The cells were then rinsed twice with PBS at room temperature and subjected to centrifugation at 400× *g* for 5 min at room temperature. After removing the supernatant, the cell pellets were resuspended in 200 µL of PBS at room temperature for 20 min. Next, 0.4 µL of Cyto-ID Green stain solution was added, and the cells were stained for 5 min at room temperature. This was followed by the addition of 0.2 µL of Hoechst 33342 stain solution for a 20 min incubation. The autophagic flux was quantified by collecting the cells and staining them with Cyto-ID Green fluorescent dye, which was detected at an excitation wavelength of approximately 480 nm and an emission wavelength of approximately 530 nm.

### 2.12. Interleukin 1 Beta (IL-1β) Measurement

The cellular IL-1β level was assessed using the ELISA assay (#ADI-900-130A, Enzo Life Sciences, Farmingdale, NY, USA) according to the manufacturer’s guidelines. In brief, homogenized cell lysates (50 µL) were incubated with 100 µL of human IL-1β biotin conjugate solution in wells coated with human IL-1β antibody on a 96-well strip-well plate at room temperature for 2 h. A standard curve was generated using different concentrations of a human IL-1β standard (ranging from 0 to 250 pg/mL). Following washing steps, 100 µL of streptavidin–peroxidase substrate solution was added to each well (excluding the chromogen blanks) and incubated for 30 min at room temperature. The reaction was halted by adding 100 µL of stop solution to each well. Optical densities were measured at 450 nm using a microplate reader within 2 h after the addition of the stop solution. IL-1β concentrations were determined by comparing the optical densities to the standard curve generated using GraphPad Prism 8 software (GraphPad Software Inc., San Diego, CA, USA).

### 2.13. Western Blotting

Cells were harvested and lysed using RIPA lysis and extraction buffer (#89900, Thermo Fisher Scientific, Waltham, MA, USA) supplemented with 1× Halt Protease and Phosphatase Inhibitor Cocktail (#78441, Thermo Fisher Scientific). The protein concentration in the total cell lysates was determined using Pierce BCA Protein Assay Kits (#89900, Thermo Fisher Scientific) following the manufacturer’s protocol. Subsequently, the protein samples were separated on an SDS-PAGE gel and transferred onto a PVDF membrane (GE Healthcare-Amersham Biosciences, Chicago, IL, USA) using a semi-dry transfer system (Bio-Rad, Hercules, CA, USA). The membrane was then blocked with 5% skim milk in TBST for 45 min at room temperature. After overnight incubation with primary antibodies (refer to Table S1) at 4 °C in skim milk, the membrane was washed three times in TBST for 10 min each and incubated with secondary antibodies (refer to Table S1) in TBST for one hour. Following three additional washes with TBST for 10 min each, the signal was detected using an Enhanced Chemiluminescence (ECL) detection system (#34080, Thermo Fisher Scientific). Protein quantification was performed using ImageJ^®^ software (Version 1.53e, NIH, Bethesda, MD, USA). The graphical representations depict the mean values (±SD) derived from at least three independent experiments.

### 2.14. Statistical Analysis

All experimental data were analyzed using the GraphPad Prism 8 software program (San Diego, CA, USA) based on the number of replicates and group design. Ordinary one-way ANOVA or two-way ANOVA with Tukey’s multiple comparisons test was employed for the analysis, as specified in each figure legend.

## 3. Results

### 3.1. Effects of H_2_O_2_ on Human Nucleus Pulposus Cells (hNPCs) Growth, Stress-Responsive ROS, Autophagy, and Cell Death

**Figure 1:**
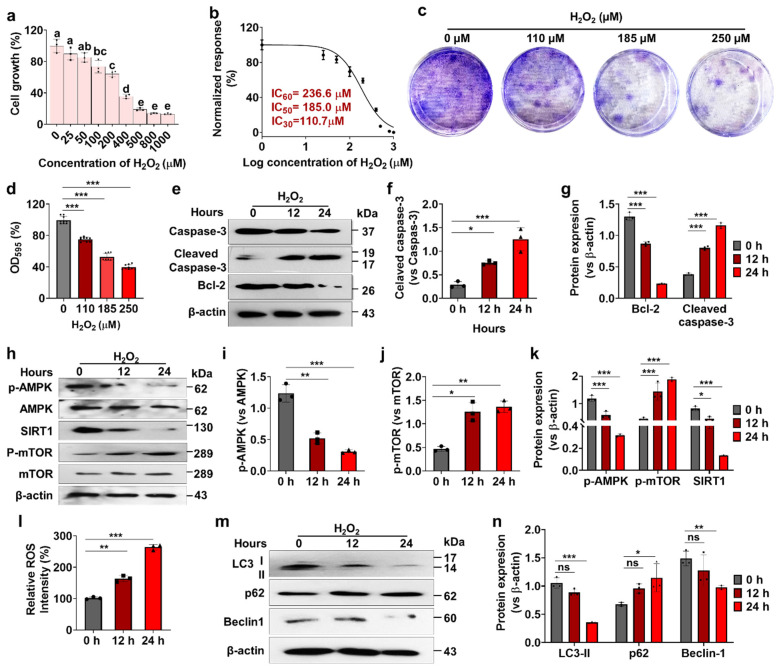
H_2_O_2_-induced oxidative stress decreases cell viability, increases ROS generation and cell death, and manipulates AMPK signaling and autophagy flux. (**a**) Cell growth rate following H_2_O_2_ treatment on hNPCs was assessed using the CCK-8 assay. Distinct letters indicate statistically significant variances. (**b**) The inhibitory concentration (IC) of H_2_O_2_ was determined to assess its cytotoxic effect on cells. (**c**) Clonogenic assay was employed to evaluate proliferative potential. (**d**) Proliferation ability was quantified using crystal violet by measuring absorbance at 590 nm. (**e**) Western blot analysis was conducted to detect protein levels of caspase-3, cleaved caspase-3, and Bcl-2 proteins. (**f**) Cleaved caspase-3/caspase-3 ratio. (**g**) Expression level of cleaved caspase-3 and Bcl-2 relative to β-actin. (**h**) Western blot analysis was performed to detect protein levels of p-AMPK, AMPK, SIRT1, p-mTOR, and mTOR. (**i**) p-AMPK/AMPK ratio. (**j**) p-mTOR/mTOR ratio. (**k**) Expression level of p-AMPK, SIRT1 and p-mTOR relative to β-actin. (**l**) Fluorescence intensity was analyzed using a fluorescence microplate spectrophotometer. (**m**) Western blot analysis was conducted to detect protein levels of LC-3I/II, p62, and Beclin-1. (**n**) Expression level of LC-3II, p62, and Beclin-1 relative to β-actin. h: hours. Values are presented as mean ± SD (*n* = 3 or >3), and statistical significance was determined using ordinary one-way ANOVA with Tukey’s multiple comparisons in (**d**,**f**,**i**,**j**,**l**) and two-way ANOVA with Tukey’s multiple comparisons in (**g**,**k**) and (**n**). * *p* < 0.01 ** *p* < 0.001, and *** *p* < 0.0001 were considered statistically significant. ns: not significant.

A significant decrease in cell viability was observed with H_2_O_2_ concentrations of 100 µM or higher after 24 h of incubation. H_2_O_2_ treatment (100–1000 µM) resulted in a significant reduction in hNPC cell viability (30–85%) compared to controls (Figure 1a). The IC_30_, IC_50_, and IC_60_ values of H_2_O_2_ for hNPCs were determined to be 110.7 µM, 185.0 µM, and 236.6 µM, respectively (Figure 1b). Exposure to 185 µM H_2_O_2_ led to increased ROS production between 12 and 24 h, suggesting the presence of oxidative stress (Figure 1c). These results indicate that H_2_O_2_ treatment significantly impaired hNPC proliferation, as corroborated by the in vitro clonogenic assay (Figure 1d,e). Furthermore, treatment with H_2_O_2_ significantly elevated the expression of apoptotic proteins, cleaved caspase-3, and reduced the expression of the anti-apoptotic protein, Bcl-2, compared to controls (Figure 1f) during the 12 to 24-h period. Additionally, our findings revealed significant alterations in the AMPK pathway following H_2_O_2_ treatment, as evidenced by the upregulation of p-AMPK and SIRT1, and downregulation of p-mTOR protein expression between 12 and 24 h (Figure 1g). Moreover, H_2_O_2_ treatment significantly suppressed autophagy flux, as indicated by reduced expression of LC3-II and Beclin-1, and increased p62 protein expression between 12 and 24 h (Figure 1h).

Overall, these results highlight the induction of oxidative stress and apoptosis, as well as alterations in cellular pathways including autophagy and AMPK signaling in hNPCs upon exposure to H_2_O_2_. This finding underscores the significance of oxidative stress in the biology of hNPCs and suggests potential therapeutic strategies for intervertebral disc degeneration associated with oxidative stress.

### 3.2. Effects of Delp on H_2_O_2_-Induced Oxidative Stress in hNPCs

**Figure 2:**
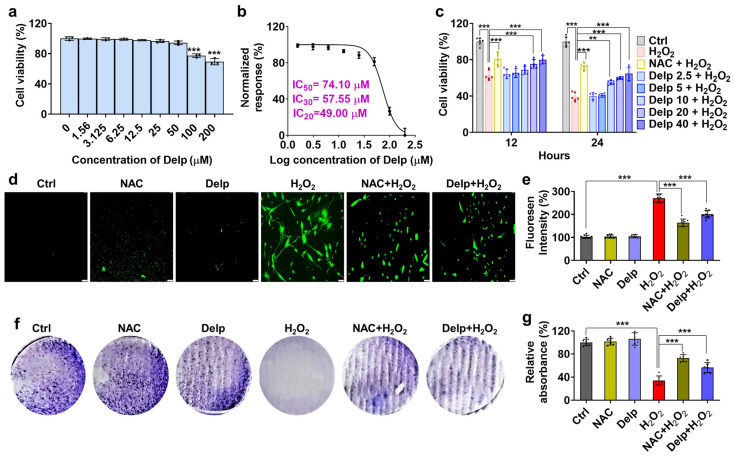
Delp increases cell viability and reduces oxidative stress in H_2_O_2_-treated hNPCs. (**a**) The cell growth rate following Delp treatment on hNPCs was assessed using the CCK-8 assay. (**b**) The inhibitory concentration (IC) of Delp was determined to identify an effective and safe dose for hNPCs. (**c**) Cell viability was evaluated using the CCK-8 assay with different doses of Delp (2.5, 5, 10, 20, and 40 µM) and 5 mM of NAC in 185 µM of H_2_O_2_ treated hNPCs at 12 and 24 h. (**d**) 2′,7′-dichlorodihydrofluorescein diacetate (DCFH2-DA) green fluorescence staining was performed to evaluate oxidative stress-induced ROS activity, with a scale bar of 50 µm. (**e**) Fluorescence intensity was analyzed using a fluorescence microplate spectrophotometer. (**f**) The clonogenic assay was utilized to assess proliferative potential. (**g**) Proliferation ability was quantified using crystal violet solution by measuring absorbance at 590 nm. Values are presented as mean ± SD (*n* = 3 or >3), and statistical significance was determined using ordinary one-way ANOVA with Tukey’s multiple comparisons in (**a**,**e**,**g**), and two-way ANOVA with Tukey’s multiple comparisons in (**c**). ** *p* < 0.001, and *** *p* < 0.0001 were considered statistically significant.

Delphinidin was administered to hNPCs at various concentrations for 24 h, and its cytotoxic effect on hNPCs was assessed using the crystal violet assay. As depicted in Figure 2a, Delp significantly reduced hNPC viability at concentrations exceeding 100 µM, while showing no effect at concentrations below 50 µM. The IC_20_, IC_30_, and IC_50_ values of Delp in hNPCs at 24 h were determined to be 49.00 µM, 57.55 µM, and 74.10 µM, respectively (Figure 2b). These values offer crucial insights into the potency of Delp in modulating hNPC viability, thereby guiding further investigations into its mechanism of action and potential therapeutic applications. Given that H_2_O_2_ concentrations above 100 µM decreased hNPC activity, with inhibition increasing with higher concentrations (Figure 1a), we selected 185 µM H_2_O_2_ for pretreatment in combination with 5 mM NAC and 2.5, 5, 10, 20, and 40 µM Delp. Figure 2c demonstrates that doses of 20 and 40 µM Delp were effective at 12 and 24 h, with 10 µM also showing efficacy at 24 h. Remarkably, ROS production increased significantly at 185 µM H_2_O_2_ (≥2.5-fold), while Delp pretreatment led to a substantial reduction (30 to 40%) in H_2_O_2_-induced ROS levels (Figure 2d,e). Furthermore, while H_2_O_2_ treatment decreased the cell survival rate of hNPCs, Delp treatment effectively rescued their clonogenic survival ability (Figure 2f,g). These findings underscore the potential of Delp in mitigating the adverse effects of H_2_O_2_ on hNPCs, highlighting its prospective therapeutic utility in oxidative stress-related conditions.

### 3.3. Confirmation of H_2_O_2_-Treated Oxidative Stress-Induced Senescence (OSIS) Model in hNPCs

**Figure 3:**
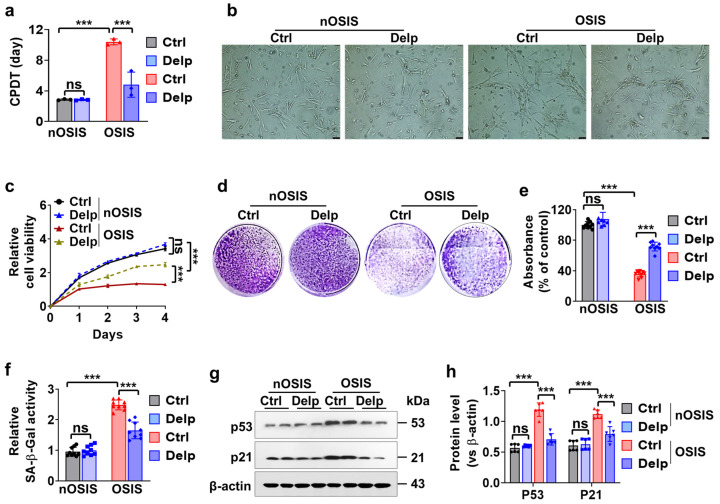
Delp induces cell proliferation and inhibits cellular senescence in OSIS hNPCs. (**a**) Cell population doubling time (CPDT) was determined to assess cellular aging. (**b**) Morphological evaluation was conducted under light microscopy, with a scale bar of 50 µm. (**c**) Relative cell viability was measured using the CCK-8 assay over a 4-day period. (**d**) The clonogenic assay was performed to evaluate proliferative potential. (**e**) The absorbance percentage of the blue-violet color at 590 nm was measured in the crystal violet assay. (**f**) Senescence activity was assessed using the senescence-associated-β-galactosidase (SA-β-gal) kit. (**g**) Western blot analysis was employed to evaluate the protein levels of p53 and p21. (**h**) Quantification of p53 and p21 protein levels relative to β-actin was conducted. Values are presented as mean ± SD (*n* = 3 or >3), and statistical significance was determined using ordinary one-way ANOVA with Tukey’s multiple comparisons in (**a**,**c**,**e**,**f**), and two-way ANOVA with Tukey’s multiple comparisons in (**h**). *** *p* < 0.0001 were considered statistically significant. ns: not significant.

We established an H_2_O_2_-induced oxidative stress-induced senescence (OSIS) model, which could serve as a valuable tool for comprehending the impact of Delp on cell growth and potentially studying intervertebral disc degeneration characterized by similar alterations in cell proliferation rates. In the OSIS model, we observed an increase in Cell Population Doubling Time (CPDT) (Figure 3a). Compared to the nOSIS control group, the OSIS control group exhibited a significant approximately 3- to 4-fold increase in CPDT (2.84 ± 0.10 vs. 10.40 ± 0.39 days, Figure 3a). Intriguingly, Delp substantially inhibited H_2_O_2_-induced CPDT by approximately 2-fold compared to the control (4.80 ± 1.69 vs. 10.40 ± 0.39 days, Figure 3a) in the OSIS model. However, when administering Delp in the nOSIS model, we did not observe any differences in CPDT compared to the control group (2.82 ± 0.08 vs. 2.84 ± 0.10 days, Figure 3a).

Next, we examined the morphological changes of hNPCs in both nOSIS and OSIS models. In the nOSIS model, hNPCs displayed a spindle-shaped morphology, characteristic of actively proliferating cells (Figure 3b). Conversely, in the OSIS model, hNPCs underwent morphological alterations, transitioning from a spindle-like shape to enlarged, flattened, and irregular shapes, indicative of reduced cell growth rates (suggesting cellular senescence). Notably, Delp treatment significantly impeded this transition and maintained a spindle-like shape (Figure 3b).

Furthermore, the relative cell viability was notably reduced in the OSIS model, whereas Delp treatment significantly restored cell proliferation activity (Figure 3c). OSIS hNPCs exhibited substantially lower proliferation rates, as evidenced by diminished clonogenic cell growth (Figure 3d), indicating cellular senescence. Delp treatment significantly ameliorated the clonogenic survival capacity of OSIS hNPCs (Figure 3d,e).

Moreover, senescence-associated β-galactosidase (SA-β-Gal) activity was higher in the OSIS model, while Delp treatment resulted in lower SA-β-Gal activity (Figure 2f). SA-β-Gal is a widely used marker of cellular senescence, reflecting the presence of senescent cells. Additionally, we evaluated the expression levels of two key senescence-related proteins, p53 and p21, in OSIS hNPCs. Both p53 and p21 are established regulators of cellular senescence pathways. Western blot analysis revealed elevated expression of senescence markers, including p53 and p21, in H_2_O_2_-induced hNPCs (Figure 3g, h). Conversely, Delp pretreatment markedly reduced the expression of these markers (Figure 3g,h). 

### 3.4. Delp Protects hNPCs from OSIS via Controlling ROS Production, ECM Synthesis, and Autophagy

**Figure 4:**
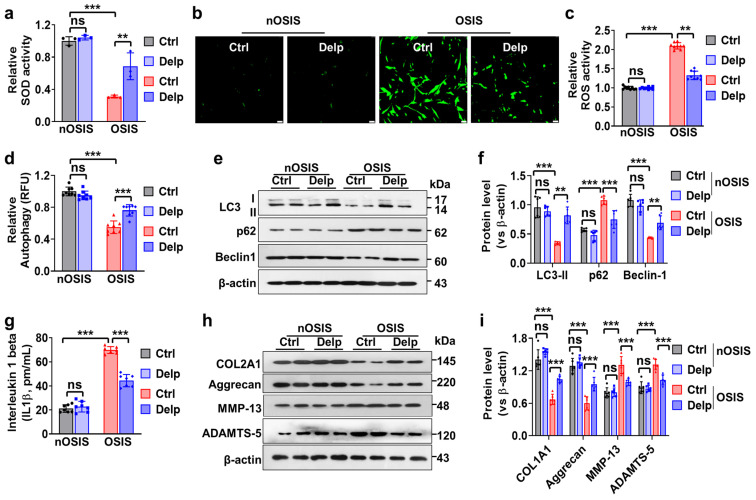
Delphinidin reduces oxidative stress and ECM degradation with increasing autophagy flux in OSIS hNPCs. (**a**) SOD activity was assessed to evaluate the antioxidant properties of Delp. (**b**) The green fluorescence staining of 2′,7′-dichlorodihydrofluorescein diacetate (DCFH2-DA) was performed to evaluate oxidative stress-induced ROS levels, with a scale bar of 50 µm. (**c**) Fluorescence intensity was analyzed using a fluorescence microplate spectrophotometer. (**d**) Relative autophagy flux was evaluated using an autophagy kit. (**e**) Western blot analysis was conducted to assess the expression of LC3-I/II, p62, and Beclin-1 proteins. (**f**) The expression levels of LC3-I/II, p62, and Beclin-1 proteins relative to β-actin were quantified. (**g**) The amount of interleukin 1 beta (IL-1β) was measured to evaluate IVDD phenotype in OSIS hNPCs. (**h**) Western blot analysis was performed to measure the expression of COL2A1, Aggrecan, MMP-13, and ADAMTS-5 proteins. (**i**) The expression levels of COL2A1, Aggrecan, MMP-13, and ADAMTS-5 proteins relative to β-actin were quantified. Values are presented as mean ± SD (*n* = 3 or >3), and statistical significance was determined using ordinary one-way ANOVA with Tukey’s multiple comparisons in (**a**,**c**,**d**,**g**), and two-way ANOVA with Tukey’s multiple comparisons in (**f**,**i**). ** *p* < 0.001, and *** *p* < 0.0001 were considered statistically significant. ns: not significant.

We delved deeper into the effects of Delp therapy on OSIS in hNPCs, particularly focusing on its influence on cellular responses related to oxidative stress and autophagy, as well as its impact on IVDD phenotypes. Initially, we examined how Delp affected two crucial aspects of oxidative stress: superoxide dismutase (SOD) activity and reactive oxygen species (ROS) production. SOD serves as a vital antioxidant enzyme, neutralizing superoxide radicals to safeguard cells from oxidative damage. Our findings revealed a decrease in SOD activity in OSIS hNPCs compared to nOSIS conditions (Figure 4a), indicating diminished antioxidant capacity in OSIS cells, rendering them more vulnerable to oxidative damage. Remarkably, treatment with Delp significantly enhanced SOD activity in OSIS hNPCs (Figure 4a), suggesting an augmentation in the cells’ ability to neutralize superoxide radicals, potentially mitigating oxidative stress-induced damage. Moreover, we observed ROS overproduction in OSIS hNPCs compared to nOSIS conditions (Figure 4b,c), a hallmark of oxidative stress. Notably, Delp treatment markedly attenuated ROS overproduction in OSIS hNPCs, indicating its potential to alleviate oxidative stress by reducing excessive ROS generation within the cells.

Subsequently, we investigated the role of autophagy in our senescence model, given its established role in mitigating oxidative stress. Our results revealed a significant reduction in autophagy activation in the OSIS model compared to nOSIS conditions (Figure 4d), suggesting an impaired autophagic process, possibly due to elevated oxidative stress or other cellular dysfunctions. However, treatment with Delp rescued autophagy flux in the OSIS model (Figure 4d), indicating its ability to restore or enhance the autophagic process under senescent conditions. Furthermore, we examined the expression levels of key autophagy-related proteins, LC3-I/II, Beclin-1, and p62, in OSIS hNPCs. Increasing expression levels of LC3 and Beclin-1, along with decreased expression of p62, signified significant restoration of autophagy activation in OSIS hNPCs upon Delp treatment (Figure 4e,f), suggesting that Delp-mediated rescue of autophagy flux may involve modulation of these key autophagy-related proteins.

Additionally, we explored the effect of Delp treatment on IVDD phenotypes using the OSIS model in hNPCs. We aimed to evaluate how Delp influences various molecular markers associated with IVDD. Our results demonstrated elevated levels of interleukin-1 beta (IL-1β), a pro-inflammatory cytokine implicated in IVDD pathology, in the OSIS model compared to nOSIS conditions (Figure 4g), indicative of increased inflammation associated with IVDD. However, treatment with Delp resulted in a reduction of IL-1β levels induced by oxidative stress (Figure 4g), suggesting its anti-inflammatory properties that may alleviate IVDD-related inflammation. Moreover, Western blot analysis revealed alterations in the expression levels of key ECM proteins associated with IVDD, including COL2A1, aggrecan, MMP-13, and ADAMTS-5, in the OSIS model. Delp treatment promoted COL2A1 and aggrecan expression while inhibiting MMP-13 and ADAMTS-5 expression in OSIS hNPCs (Figure 4h,i), indicating its potential to protect against IVDD by enhancing the synthesis of structural proteins and inhibiting enzymes responsible for disc degradation, thereby preserving disc integrity and function.

### 3.5. Autophagy Enhanced the Inhibitory Effect of Delp on the Senescence and ECM Degradation in OSIS hNPCs

**Figure 5:**
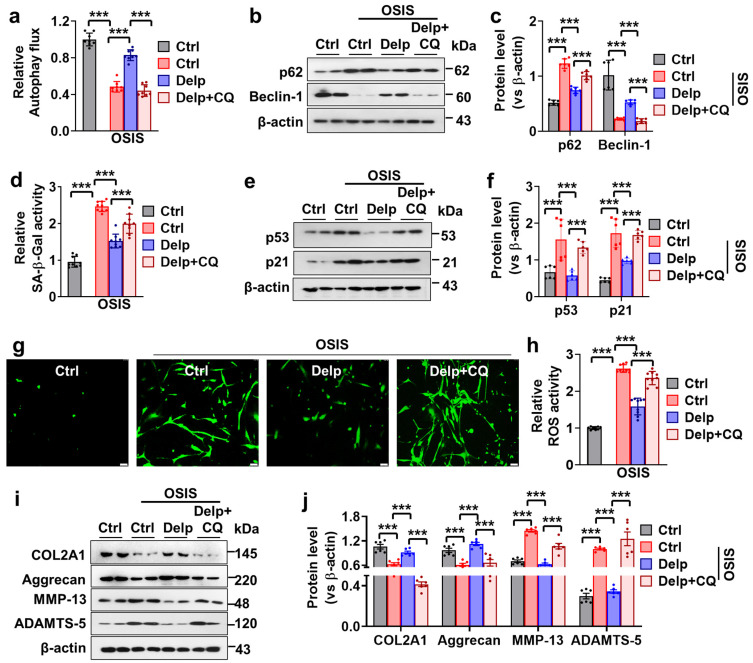
Inhibition of autophagy abolished the protective effect of Delp in OSIS hNPCs. (**a**) The reduction of Delp-induced autophagy by CQ treatment was assessed by evaluating the relative autophagy flux using an autophagy kit. (**b**) Western blot analysis was conducted to evaluate the expression levels of p62 and Beclin-1. (**c**) The expression levels of p62 and Beclin-1 proteins relative to β-actin were quantified. (**d**) Senescence activity was measured using a senescence-associated-β-galactosidase (SA-β-gal) kit. (**e**) Western blot analysis was performed to evaluate the protein levels of p53 and p21. (**f**) The expression levels of p53 and p21 proteins relative to β-actin were quantified. (**g**) The green fluorescence staining of 2′,7′-dichlorodihydrofluorescein diacetate (DCFH2-DA) was performed to evaluate oxidative stress-induced ROS levels, with a scale bar of 50 µm. (**h**) Fluorescence intensity was analyzed using a fluorescence microplate spectrophotometer. (**i**) Western blot analysis was conducted to measure the expression levels of COL2A1, Aggrecan, MMP-13, and ADAMTS-5 proteins. (**j**) The expression levels of COL2A1, Aggrecan, MMP-13, and ADAMTS-5 proteins relative to β-actin were quantified. Values are presented as mean ± SD (*n* = 3 or >3), and statistical significance was determined using ordinary one-way ANOVA with Tukey’s multiple comparisons in (**a**,**d**,**h**), and two-way ANOVA with Tukey’s multiple comparisons in (**c**,**f**,**j**). *** *p* < 0.0001 was considered statistically significant.

To confirm the involvement of autophagy in cellular senescence and extracellular matrix (ECM) degradation in OSIS hNPCs, we employed chloroquine (CQ), a known autophagy inhibitor, to block the autophagic process and observe its effects on Delp-mediated autophagy activation and associated protein expression. Initially, Delp treatment activated autophagy in OSIS hNPCs, as evidenced by increased expression of key autophagy-related proteins such as Beclin-1, alongside decrease of p62 protein (Figure 5a–c). This indicates that Delp effectively induced autophagy in OSIS hNPCs, potentially contributing to cellular protection against oxidative stress and senescence. Subsequently, co-treatment of OSIS hNPCs with CQ abolished Delp-mediated autophagy activation, as evidenced by a significant reduction in the expression of Beclin-1 and the increased level of p62 protein (Figure 5a–c), suggesting that CQ effectively inhibited the autophagic process initiated by Delp, leading to the accumulation of autophagy-related proteins and disruption of autophagic flux.

We continued our investigation into the role of autophagy in attenuating the cellular senescence of hNPCs subjected to OSIS. Inhibition of autophagy by CQ had significant consequences on several senescence-related parameters in OSIS hNPCs treated with Delp. Firstly, the attenuation of SA-β-Gal activity by Delp was reversed upon treatment with CQ (Figure 5d), indicating that the inhibition of autophagy by CQ interfered with the ability of Delp to attenuate cellular senescence. Additionally, Delp treatment effectively reduced the expression of p53 and p21 in the OSIS model, indicative of its ability to mitigate senescence-associated signaling pathways (Figure 5e,f). However, the inhibitory effect of Delp on p53 and p21 expression was significantly attenuated by CQ treatment, suggesting that the inhibition of autophagy compromises Delphinidin regulation of senescence-related protein expression in OSIS hNPCs (Figure 5e,f).

Furthermore, we explored how autophagy inhibition influenced the effects of Delp on ROS production in hNPCs subjected to OSIS. Delp treatment significantly reduced ROS overproduction in OSIS hNPCs, indicating its antioxidant properties and ability to mitigate oxidative stress-induced damage (Figure 5g,h). However, co-administration of CQ with Delp reversed the effect of Delp on ROS production (Figure 5g,h), suggesting that autophagy inhibition interferes with Delp’s antioxidant mechanisms, leading to the restoration of ROS overproduction in OSIS hNPCs.

Finally, we investigated the impact of autophagy inhibition on the effects of Delp on IVDD phenotypes in OSIS hNPCs. Delp treatment promoted the expression of ECM synthesis proteins, such as COL2A1 and aggrecan, while inhibiting the expression of ECM degradation proteins, including MMP-13 and ADAMTS-5, in the OSIS model (Figure 5i,j), indicating its potential to promote ECM homeostasis and protect against IVDD. However, co-administration of Delp and CQ reversed the Delp-mediated effects on ECM homeostasis (Figure 5i,j), suggesting that autophagy inhibition interfered with Delp-dependent regulation of ECM synthesis and degradation proteins in OSIS hNPCs.

### 3.6. Activation of the AMPK Pathway Is Important for Delp-Induced Autophagy in the OSIS hNPCs

**Figure 6:**
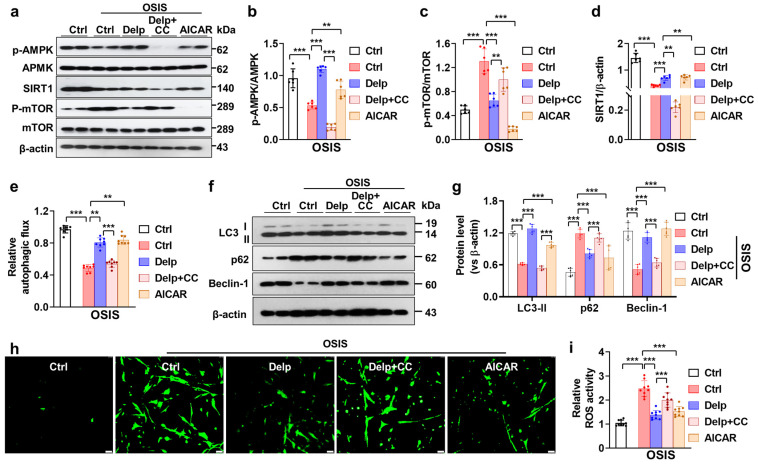

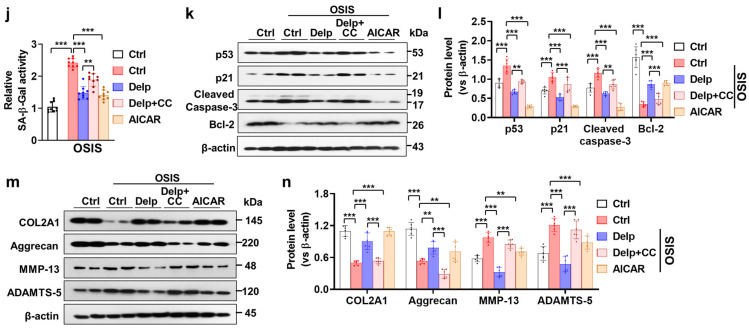
The AMPK pathway mediates Delp-induced autophagy in OSIS hNPCs. (**a**) Protein levels of p-AMPK, AMPK, SIRT1, p-mTOR, and mTOR were assessed using Western blot analysis. (**b**) The ratios of phospho-AMPK to AMPK were calculated. (**c**) The ratios of phospho-mTOR to mTOR were determined. (**d**) The expression level of SIRT1 relative to β-actin was quantified. (**e**) The relative autophagy flux was measured to assess the effect of CC on Delp-induced autophagy. (**f**) Western blot analysis was performed to evaluate the expression levels of LC3-I/II, p62, and Beclin-1. (**g**) The expression levels of LC3-II, p62, and Beclin-1 relative to β-actin were quantified. (**h**) 2′,7′-dichlorodihydrofluorescein diacetate (DCFH2-DA) staining was used to evaluate oxidative stress-induced ROS, with a scale bar of 50 µm. (**i**) Fluorescence intensity was analyzed using a fluorescence microplate spectrophotometer. (**j**) Senescence activity was measured using a senescence-associated-β-galactosidase (SA-β-gal) kit. (**k**) Western blot analysis was conducted to evaluate the protein levels of p53, p21, cleaved caspase-3, and Bcl-2. (**l**) The expression levels of p53, p21, cleaved caspase-3, and Bcl-2 relative to β-actin were quantified. (**m**) Protein levels of COL2A1, Aggrecan, MMP-13, and ADAMTS-5 were assessed using Western blot analysis. (**n**) The expression levels of COL2A1, Aggrecan, MMP-13, and ADAMTS-5 relative to β-actin were quantified. Values are presented as mean ± SD (*n* = 3 or >3), and statistical significance was determined using ordinary one-way ANOVA with Tukey’s multiple comparisons in (**b**–**e**,**i**,**j**), and two-way ANOVA with Tukey’s multiple comparisons in (**g**,**l**,**n**). ** *p* < 0.001, and *** *p* < 0.0001 were considered statistically significant.

Based on our findings, it appears that treatment with chloroquine (CQ) reduces the autophagy induced by Delp in OSIS hNPCs, suggesting the crucial role of Delp-induced autophagy in its protective effect. We further explored the potential mechanisms underlying the autophagy-inducing effect of Delp in OSIS hNPCs. AMPK is recognized for its role in governing protective autophagy, enabling cells to counteract diverse stressors. By phosphorylating autophagy-related protein complexes, AMPK actively promotes autophagy across various regulatory stages. We hypothesized that Delp triggers autophagy in OSIS cells through activation of the AMPK signaling pathway. To investigate this hypothesis, we utilized compound C (CC), a potential inhibitor of AMPK, and AICAR, an analog of AMPK. As expected, like AICAR, Delp remarkably activated phosphorylated AMPK (p-AMPK) and SIRT1 proteins, while reducing phosphorylated mTOR (p-mTOR) protein expression in the OSIS hNPCs model (Figure 6a–d). Furthermore, Delp-mediated SIRT1/AMPK-mTOR activation was negated by CC treatment. Notably, the induction of autophagy by Delp was significantly diminished with CC treatment, evidenced by decreased levels of LC3-I/II and Beclin-1, along with elevated p62 protein expression in OSIS hNPCs (Figure 6e–g). These results strongly suggest that Delp-induced AMPK activation leads to autophagy activation in OSIS hNPCs. Moreover, our data revealed that AMPK activation by either AICAR or Delp notably reduced the overproduction of reactive oxygen species (ROS), whereas CC treatment increased ROS production suppressed by Delp (Figure 6h,i). Moreover, inhibition of the AMPK signaling pathway by CC markedly induced the attenuation of SA-β-gal activity in OSIS hNPCs (Figure 6j). Correspondingly, Western blot data indicated that CC treatment reversed the effects of Delp on the expression of senescence proteins p53 and p21 in the OSIS model (Figure 6j,k). Additionally, the Western blot analysis of IVDD candidate proteins showed significant reductions in COL2A1 and Aggrecan in the Delp plus CC-treated group, alongside increases in MMP-13 and ADAMTS-5 (Figure 6l,m). These findings underscore the critical role of AMPK signaling in mediating the effects of Delp on autophagy, ROS production, and senescence in OSIS hNPCs, highlighting its potential as a therapeutic target for IVDD. Taken together, our findings indicate that Delp activates autophagy through AMPK and reduces oxidative stress-induced senescence and IVDD phenotypes.

## 4. Discussion

Our study demonstrated that Delp exerts antioxidant effects by mitigating ROS-induced oxidative stress and cellular senescence in human IVD NPC cells. Moreover, Delp simultaneously induces autophagy, fostering cell growth and ECM synthesis while suppressing ROS overproduction and cellular senescence. Inhibition of autophagy using CQ in hNPCs diminished the protective effect of Delphinidin on ROS overproduction, cellular senescence, and ECM degradation. This protective autophagy induction by Delp is mediated via the activation of the SIRT1/AMPK-mTOR signaling pathway in hNPCs.

Hydrogen peroxide (H_2_O_2_) was selected as a ROS inducer to simulate an in vitro oxidative stress condition in our study. We established an “H_2_O_2_-induced OSIS” model to investigate the effects of Delp on cell growth under oxidative stress conditions relevant to IVDD. This model is tailored to mimic oxidative stress-induced conditions in IVDD, providing insights into how Delp influences cell growth under such circumstances.

Oxidative stress, driven by ROS generation, triggers redox imbalance and lipid peroxidation, contributing to cellular cytotoxicity when exceeding the cell’s repair capacity [26]. In degenerative disorders lacking sufficient antioxidant defense mechanisms, ROS production substantially disrupts cell homeostasis, leading to the accumulation of damaged DNA with molecular alterations [27]. Delphinidin, a polyphenol from the anthocyanin group, has been shown in previous studies to protect against age-related oxidative stress, playing a critical role in preventing osteoarthritis development and progression [28,29,30,31]. Consistent with prior reports, our study demonstrated the cytoprotective effect of Delp against oxidative stress, highlighting its potential as a clinical cytoprotective agent.

Prolonged ROS release induces oxidative stress, triggering premature senescence, apoptosis, and eventual cell death [32]. In our study, Delp modulated SA-β-gal enzymatic activity and regulated the expression of senescence-related proteins p53 and p21, mediating cell growth through a cytoprotective pathway. Autophagy initiation has been suggested to protect against apoptosis in IVDD, underscoring its cytoprotective role [33,34]. While current research predominantly supports autophagy’s preventive role in IVDD, some studies suggest its potential to accelerate the condition [35]. Autophagy reduces NP cell apoptosis, ECM degradation, senescence, and CEP inflammation and calcification, maintaining IVD ECM balance to prevent IVDD [36,37,38,39,40].

Numerous signaling pathways regulate autophagy induction, with AMP-activated protein kinase (AMPK) being a key player impacted by ROS. AMPK serves as a significant indicator of cellular energy status and a central regulator of autophagy and lysosomal activity [41]. Several natural compounds regulate autophagy through the AMPK pathway in NP cells against oxidative stress-induced senescence, ECM degradation, and apoptosis [42,43,44]. Our study explored the molecular mechanism of Delp-induced autophagy and its relationship with the AMPK pathway. Delp upregulated SIRT1 function upstream of AMPK phosphorylation, affecting mTOR phosphorylation downstream of AMPK, thereby rescuing autophagy. Compound C, an AMPK inhibitor, notably reduced Delp-mediated autophagy activation [45]. This highlights its protective effect on hNPCs through the AMPK pathway, linking cell proliferation and autophagy induction. Further in vivo experiments are warranted to validate these findings.

## 5. Conclusions

**Figure 7:**
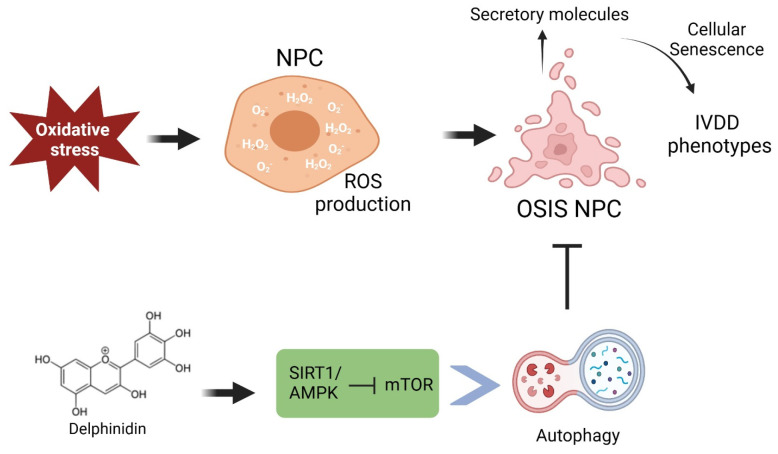
The proposed molecular mechanism involved in Delphinidin treatment in OSIS hNPCs. Hydrogen peroxide (H_2_O_2_) serves as a source of reactive oxygen species (ROS), inducing oxidative stress conditions and mimicking intervertebral disc degeneration (IVDD) phenotypes in human nucleus pulposus cells (hNPCs), leading to a state of oxidative stress-induced senescence (OSIS). Delphinidin (Delp) mitigated OSIS in H_2_O_2_-exposed hNPCs by enhancing cellular autophagy through activation of the SIRT1/AMPK/mTOR axis.

This study presents the initial evidence suggesting that Delp has the potential to safeguard hNP cells against senescence, apoptosis, and ECM degradation induced by oxidative stress-driven ROS overload by activating autophagy. This protective effect is likely mediated through the ROS–AMPK–mTOR axis, as illustrated in Figure 7. The emerging therapeutic role of Delp in combating oxidative stress in hNPCs could pave the way for novel treatment strategies of IVDD. Nevertheless, the exact mechanism underlying Delphinidin action requires further investigation, and these findings need validation in in vivo settings.

## Data Availability

Data is contained within the article and Supplementary Materials.

## Supplementary Materials

The following supporting information can be downloaded at: https://www.mdpi.com/article/10.3390/antiox13070759/s1.
