# Supplementary material for: The Survival of Human Intervertebral Disc Nucleus Pulposus Cells under Oxidative Stress Relies on the Autophagy Triggered by Delphinidin
Source: Antioxidants (Basel). 2024 Jun 23;13(7):759. doi: 10.3390/antiox13070759 (PMC11273539; doi:10.3390/antiox13070759)
Supplement: Supplementary file 1 [file antioxidants-13-00759-s001.zip › antioxidants-3056672-supplementary.pdf]

antioxidants

MDPI

Supplementary File

Article

The survival of human intervertebral disc nucleus pulposus cells under oxidative stress relies on the autophagy triggered by delphinidin

Md Entaz Bahar[1], Jin Seok Hwang[1], Trang Huyen Lai[1], June-Ho Byun[2], Dong-Hee Kim[3,*] and Deok Ryong Kim[1,*]

[1]Department of Biochemistry and Convergence Medical Sciences and Institute of Medical Science, Gyeongsang National University College of Medicine, Jinju, Republic of Korea
[2]Department of Oral and Maxillofacial Surgery and Institute of Medical Science, Gyeongsang National University College of Medicine and Gyeongsang National University Hospital, Jinju, Republic of Korea
[3]Department of Orthopaedic Surgery and Institute of Medical Science, Gyeongsang National University College of Medicine and Gyeongsang National University Hospital, Jinju, Republic of Korea
*Correspondence: Dong-Hee Kim (dhkim8311@gnu.ac.kr), Deok Ryong Kim (drkim@gnu.ac.kr).

antioxidants

MDPI

Supplementary Figures

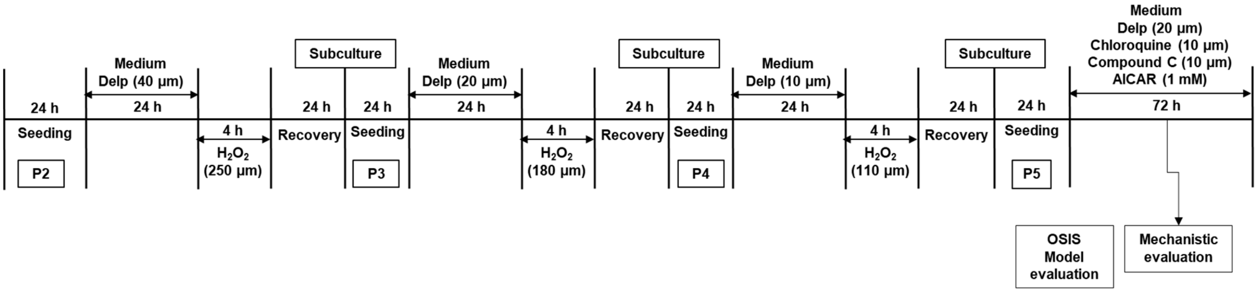

Figure S1: A flow diagram of the establishment of oxidative stress-induced senescence (OSIS). The hNPCs were cultured in NPCM with or without Delp, and then exposed to varying concentrations of H_2O_2 (250, followed by 185 and 110 µM) across passages (P2 to P5). Senescence was confirmed through assays. The mechanistic evaluation involved culturing senescence cells with normal media or treatments such as Delp, chloroquine, AICAR, or compound C for 72 h.

antioxidants

MDPI

Supplementary Tables

Table S1. List of antibodies used in western blot

Antibodies	Target	Host	Clonality	Dilution	Catalog #	Company
**Primary antibody**	COL2A1	Rabbit	Polyclonal	1:500	ABP0074	Abbkine
	Aggrecan	Mouse	Monoclonal	1:500	sc-33695	Santa Cruz
	MMP-13	Mouse	Monoclonal	1:500	sc-515284	Santa Cruz
	ADAMTS-5	Rabbit	Polyclonal	1:1000	ab182795	Abcam
	Cleaved caspase-3	Rabbit	Polyclonal	1:1000	9661	Cell Signaling
	Caspase-3	Rabbit	Polyclonal	1:500	9662	Cell Signaling
	Bcl-2	Mouse	Monoclonal	1:500	sc-7382	Santa Cruz
	p21	Mouse	Monoclonal	1:500	sc-6246	Santa Cruz
	p53	Mouse	Monoclonal	1:500	sc-126	Santa Cruz
	LC3-I/II	Rabbit	Monoclonal	1:1000	12741	Cell Signaling
	p62	Rabbit	Polyclonal	1:1000	5114	Cell Signaling
	Beclin-1	Rabbit	Monoclonal	1:1000	3495	Cell Signaling
	AMPKα	Rabbit	Polyclonal	1:1000	2532	Cell Signaling
	p-AMPKα (Thr172)	Rabbit	Monoclonal	1:2000	4188	Cell Signaling
	mTOR	Rabbit	Polyclonal	1:500	2972	Cell Signaling
	p-mTOR (Ser2448)	Rabbit	Polyclonal	1:500	2971	Cell Signaling
	SIRT1	Rabbit	Monoclonal	1:1000	9475	Cell Signaling
	β-actin	Mouse	Monoclonal	1:500	sc-47778	Santa Cruz
**Secondary antibody**	Mouse IgG	Goat	Polyclonal	1:10000	STAR117P	Bio-Rad
	Rabbit IgG	Goat	Polyclonal	1:10000	STAR208P	Bio-Rad
